# Experiences of implementing the ‘Making Every Contact Count’ initiative into a UK integrated care system: an interview study

**DOI:** 10.1093/pubmed/fdad173

**Published:** 2023-09-17

**Authors:** Rebecca Turner, Lucie Byrne-Davis, Panayiotis Michael, Nia Coupe, Caroline Holtom, Cheryl Smith, Jo Hart

**Affiliations:** Division of Psychology and Mental Health, Faculty of Biology, Medicine & Health, The University of Manchester, Manchester M13 9PL, UK; Division of Medical Education, Faculty of Biology, Medicine & Health, The University of Manchester, Manchester M13 9PLG, UK; Division of Medical Education, Faculty of Biology, Medicine & Health, The University of Manchester, Manchester M13 9PLG, UK; Division of Psychology and Mental Health, Faculty of Biology, Medicine & Health, The University of Manchester, Manchester M13 9PL, UK; Public Health North West, NHS England, North West, London L3 4BL, UK; Public Health, Lancashire County Council, Lancashire L39 2DF, UK; Division of Medical Education, Faculty of Biology, Medicine & Health, The University of Manchester, Manchester M13 9PLG, UK

**Keywords:** COM-B model, healthcare professionals, implementation, integrated care system, Make Every Contact Count, qualitative

## Abstract

**Background:**

The ‘Making Every Contact Count’ (MECC) approach is in line with the current National Health Service (NHS) strategy to improve and prevent health conditions in England. Despite its importance and value for preventative healthcare, implementation of MECC varies. The aim of this study was to explore the barriers and facilitators of implementing MECC and MECC training into an integrated care system (ICS).

**Methods:**

Remote semi-structured interviews were conducted with staff across an ICS in the North West of England who were involved in implementing and delivering MECC across the region. Data were analysed initially using an inductive thematic analysis approach and then interpreted using the ‘Capability, Opportunity, Motivation = Behaviour’ (COM-B) model of behaviour change.

**Results:**

We interviewed nine stakeholders and identified three superordinate themes: (1) macro-level barriers and facilitators, e.g. funding; (2) organizational level barriers and facilitators, e.g. time and resource; and (3) individual-level barriers/facilitators for both MECC trainers and MECC agents.

**Conclusions:**

MECC has potential to meet the needs of the public’s health, but barriers to its implementation exist. MECC must be successfully embedded into organizations and regions in which it is implemented, which relies on further development of an appropriate infrastructure including sustainable funding and a shift in culture to value preventative healthcare.

## Introduction

Making Every Contact Count (MECC) is a national initiative and evidence-based approach to improve people’s health and well-being,^1^ in response to the National Institute for Health and Clinical Excellence (NICE) guidance on promoting health-related behaviour change.^2^ The MECC approach involves initiating a very brief intervention, with a person as part of a routine appointment or consultation.^1^^,^^3^^,^^4^ Offering appropriate advice, raising awareness of risks, providing encouragement and support for change, and/or signposting and referring them to local services/sources of further information are some of the typical activities that would take place in these discussions.^1^^,^^3^^,^^4^

MECC has the potential to deliver a significant public health resource, at a low cost, across a variety of healthcare contexts to improve public health.^5–7^ Despite MECC being well cited by policymakers, a survey in 2018 reported that only 31% of healthcare professionals (HCPs) have heard of the approach and when a need to deliver a MECC intervention was identified by staff, it was only delivered on half of these occasions.^8^ An initial evaluation of MECC in 2013 utilized in-depth interviews with key stakeholders from a range of different organizations and found that whilst the responses were all positive, there had been variability in the take up across different organizations.^5^ Whilst they identified that MECC’s aims and objectives aligned well with many organizations, uptake had been lower in some health services due to a lack of resources, tensions between preventative approaches and managing current rates of illness within healthcare, and the cultural view that MECC was viewed as an add-on of care and not essential.^6^ This led to great variability in the delivery of MECC training across organizations.^6^ Often research has investigated health and social care workers’ perspectives on delivering MECC, and whilst considering the views of health workers is important, often the wider perspectives of implementation can be forgotten, leading to interventions not being adopted into practice.^9^

There is a need to explore different models of implementation of MECC to better understand the barriers, facilitators and the future potential for this approach.^10^^,^^11^ Understanding these factors can be aided through applying a psychological framework^12^ such as the ‘Capability, Opportunity, Motivation = Behaviour’ (COM-B) model.^12^ The COM-B model suggests that an individual’s behaviour is influenced by their capability (such as skills and knowledge), opportunity (such as time and social pressure) and motivation (such as beliefs and habits). Models such as COM-B provide a framework for understanding the complex interactions between factors that influence behaviour change.^13^ This model in particular emphasizes the importance of considering not only individual-level factors, but also broader contextual factors that can either enable or hinder behaviour change. Therefore, using COM-B as a lens can help ensure that implementation strategies are aligned with the specific needs and contexts of individuals and communities involved, ultimately improving the chances of success and sustainability of the intervention in question. Examples of the widely used application of the COM-B model to understand implementation include understanding infection and prevention control behaviours amongst HCPs^14^ and exploring barriers and facilitators to providing diet and physical activity advice to young mothers.^15^ The aim of this study was to explore the implementation barriers and facilitators of MECC and MECC training into an integrated care system (ICS).

## The context: implementation of MECC in an integrated care system

Integrated care systems (ICS) are partnerships of organizations (e.g. hospital-based care and general practitioners) that join together to plan and deliver joined up health and care services, with the aim to improve the care and health of people. An ICS within the North West of England covering both rural and urban locations, serving a population of 1.8 million, and which includes some of the most deprived areas in the UK, funded MECC as a priority deliverable for 2018–2019, via their Local Workforce Action Board to deliver a 2-year pilot. The aim of the project was to inform, motivate and empower the people living within the ICS footprint to live healthier lives through the delivery of health messages and access to support services.

The MECC initiative in the ICS in the North West of England included the following:

1. A multidisciplinary steering group to support the programme and recruit a MECC co-ordinator to manage the programme.
2. ‘Train the Trainer’ package, for MECC trainers to train up MECC agents (health and social care workers delivering MECC initiatives in practice). This package covered the following aspects:
  - An introduction to the concept of MECC and the rationale for MECC
  - An overview of health improvement and the importance of this within the area of the ICS
  - An introduction to behaviour change and motivational interviewing
  - Information and tips about developing and delivering the MECC training, including information about learning styles
3. A workforce and public facing a communication campaign ‘Chat to Change’ to promote and endorse the MECC approach and training.
4. A developed resource website called ‘MECClink’ where resources (e.g., smoking cessation support or financial inclusion information) could be accessed by MECC trainers and agents which highlights all local services health workers could signpost to: www.MECClink.co.uk/lancashire-and-south-cumbria.

## Method

### Study design

Qualitative semi-structured interviews, guided by the Standards for Reporting Qualitative Research and Consolidated Criteria for Reporting Qualitative Research^16^^,^^17^; see Supplementary file 1.

### Participants and researchers

Convenience sampling was used in this study. Participants were stakeholders working in several areas across the MECC project, including planning and implementing MECC, delivering training, managing trainers and promoting MECC to various organizations within the ICS.

A total of 122 individuals from 62 organizations across the ICS participated in a MECC Train the Trainer programme. The organizations involved in training were in seven health and social care sectors (Clinical and Nursing; Volunteering; Local Authority and Public Health; Mental Health and Recovery; Social Prescribing; Housing and Refuge; Charities and Third Sector).

The research team (authors) was made up of multidisciplinary colleagues with expertise in public health interventions and the delivery of MECC (CH and CS), and health psychology and behavioural science (RT, JH, NC, PN and LBD). Researchers responsible for data collection and analysis were researchers employed at Higher Education Institutions, with PhD’s (NC and RT) and Master’s degrees (PN), all which had experience in qualitative methods.

### Recruitment

An initial recruitment email was sent to all key contacts at the local County Council’s Public Health MECC team to express an interest in taking part in the study. Potentially interested participants agreed for their contact details to be passed onto a member of the research team. An email was then sent to these participants with a participant information sheet outlining the purpose of the study, what procedures would take place, information about data protection and confidentiality, as well as relevant contact information. Willing volunteers responded to the email and virtual interview dates were organized. Informed consent was gained from participants at the start of each interview and recorded. Participants were asked if they had any further questions and the consent form was read out, with participants providing their consent for each of the statements on the consent form.

### Data collection

**Fig. 1:**
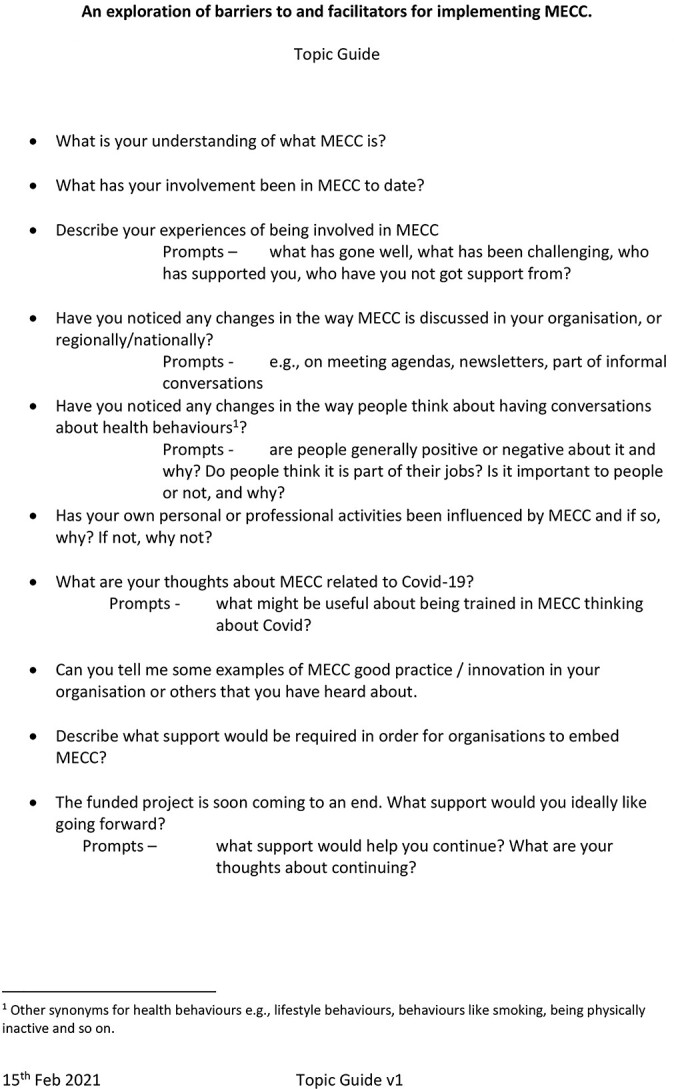
Topic guide—an exploration of barriers to and facilitators for implementing MECC.

Interviews were conducted remotely (e.g. phone, Zoom, Skype or MS Teams). Interviews were conducted by NC and PM. To the best of our knowledge, nobody else was present; however, it is not possible to verify this. Interviews followed a semi-structured topic guide (see Fig. 1), as well as follow-up questions based on participants’ responses. The topic guide aimed to understand participants’ experiences of MECC, any changes they had noticed following implementation of MECC and what further support teams felt they needed to continue with this. The topic guide was informed by the literature and the teams’ prior knowledge and experience of MECC. Interviews lasted approximately 30–40 minutes. Interviews were audio-recorded and transcribed using Otter.ai. Transcriptions were checked for accuracy and anonymized by redacting any information about people, places or organizations. Transcripts were then uploaded into NVIVO Pro 12 version. Field notes were not used and transcripts were not returned to participants for comments. Sample size was determined pragmatically by recruitment constraints. The research team aimed to recruit as many of the pool as possible and ended recruitment when the list had been exhausted. However, due to the impact of the COVID-19 pandemic, the research team did not have the opportunity to consider further recruitment strategies.

### Data analysis

Data were first analysed using an inductive approach to thematic analysis to code the data and form themes representing barriers and facilitators to implementing MECC.^18^ NC and PN began by initially reading through the transcripts and began coding. Once initial codes were made, they were gathered to form potential themes, which were reviewed by all co-authors. Subsequently, a pre-existing understanding amongst the researchers about the overarching influential factors of behaviours, i.e. capability, opportunity and motivation (Michie et al., 2011), enabled the researchers to adopt a somewhat deductive approach to the application of the themes generated to the specific research question. RT reviewed the themes in relation to the constructs of the COM-B model, assigning each theme to the COM-B model it was judged to best represent. The final analysis was presented to all co-authors and discussed with health psychologists (JH and LBD).

Analysing the data using both inductive and deductive approach allows for a rich understanding of the data and a rigorous approach to qualitative analysis.^19^ Inductive approaches are used to understand what is occurring in the data, without forcing the data into a specific theory or theoretical framework and missing potential factors.^20^ A deductive approach then enables the researcher to adopt an organized and specific approach to understanding implementation using a theoretical framework.^21^

### Ethical approval

The project was reviewed and approved by the University of Manchester Proportionate University Research Ethics Committee (Reference: 2021-11326-17879).

## Results

### Participants

Eighteen individuals were contacted to take part in the study and expressed an interest. In total, nine individuals from different sections within a UK National Health Service (NHS) ICS who had been involved in the management training and delivery of the MECC pilot took part in an interview. Please see Table 1 for an overview of participant’s roles.

**Table 1:** Overview of participant’s roles

Type of role	Number of participants (total = 9)
Public health worker/lead/doctor	4 (44%)
MECC trainer leader	1 (11%)
Service lead	2 (23%)
Mental health worker	1 (11%)
Role within a commissioning service	1 (11%)

Participants typically worked in local councils, ‘Public Health England’, NHS secondary care trusts, primary care, Commissioning services and Health Education England.

### Thematic analysis findings

Three superordinate themes were identified: (1) macro-level barriers and facilitators; (2) organizational level barriers and facilitators; and (3) individual-level barriers and facilitators for both MECC trainers and MECC agents. See Table 2 for an overview of themes.

**Table 2:** Overview of superordinate and sub-themes of barriers and facilitators to implementing MECC into a UK NHS integrated care system

Superordinate themes	Subordinate themes	COM-B domain	Quotes
Macro-level barriers and facilitators	Role and responsibility of implementing MECC into healthcare organizations	Social opportunity	*‘… There's nobody to take those decisions to support it, promote it, basically, to take ownership of it at a strategic level’ (Senior Public Health Worker, P1)*
	Financing and resourcing MECC	Physical opportunity	*‘But I think on a national level, we should be doing something about it and that's investing in it’ (MECC leader, P4)*
	Promoting the value and purpose of MECC to organizations	Psychological capability and reflective motivation	*‘… Almost needs a good marketing campaign that actually, MECC is part of the solution, MECC is part of moving forward as a society really into being stronger for any future pandemics’ (Senior Public Health Lead, P8)*
Organizational level barriers and facilitators	Need for time and resources to plan and implement MECC training	Physical opportunity	*‘… Looking at the situation now, because the funding stopped, the last training has been done, I think, certainly, now, it would be essential for the web resource to be maintained, where that time effort, resources are going to come from, I do not know’ (Role within a commissioning service, P5)*
	Culture change within organizations	Reflective motivation	*‘I think that's because we are you know, we are still very much sat in the medical model. That’s one of the problems with MECC’ (Service lead, P3)*
Individual-level barriers and facilitators for MECC trainers	MECC trainer motivation	Reflective motivation	*‘I do think there’s something about raising the profile and the desirability if you like. Now whether that's linked… Maybe we need to think about providing people if they do the MECC training they become adult educators, you know, some sort of accredited training program… I think we do need a system where there is a nationally agreed never mind regionally forum where staff can just book on, to get their MECC training, they become an accredited MECC trainer for their trust, and it means something, he's got some kudos to it… So whether that's academic learning points or whatever, but it's got some real clout. You know, it’s part of maybe advanced practice, that kind of thing’ (Senior Public Health Lead, P8)*
Individual-level barriers facilitators for MECC agents	Ability to deliver and signpost to appropriate services during MECC conversations	Physical opportunity	*‘But the key to it is the knowing where to signpost them to after that as well’ (Service lead,* P9)

### Macro-level barriers and facilitators

#### Role and responsibility of implementing MECC into healthcare organizations (social opportunity)

A lack of clarity was discussed amongst the participants as to who should be responsible for driving and implementing MECC into organizations. Having a regional MECC lead who could support the initiative, encourage and maintain engagement across healthcare organizations and provide national resources was suggested.

*‘An actual lead for MECC or an ICS lead, just someone higher up to lead the MECC, who gathers all of the national training, resources, what can be done at that level, and they would sort of just sit there, and be held to account’* (Service lead, Participant (P) 9).

The consensus amongst participants was that MECC should stem from a national approach of health behaviour change, i.e. driven by NHS England or Office for Health Improvement and Diversity encouraging the adoption of MECC.

*‘I suppose if it was in some guidance that NHS England put out, like can you demonstrate that your CCG or your area are delivering personalised care approaches…’* (Service lead, P9).

#### Financing and resourcing MECC (physical opportunity)

Participants discussed the challenges of funding within the MECC programmes, with funding only being available for a period of time for each project, therefore influencing the sustainability of MECC.

*‘I think one of the perpetual problems with Making Every Contact Count tends to be the kind of silo way of working… so seeking pots of money to do a particular project, similar to this piece of work, so you can piece it, you know, pieces, a bit of money for a given period of time’* (Senior Public Health lead, (Participant) P8).

Supporting MECC financially through sustained funding and providing standardized resources such as training materials would facilitate organizations to embed MECC and enable trainers to deliver the training sessions to MECC agents. Having long-term financial support from national organizations or having access to free training was suggested to increase the likelihood of organizations implementing MECC successfully.

*‘But I think on a national level, we should be doing something about it. And that's investing in it’* (MECC leader, P4).

#### Promoting the value and purpose of MECC to organizations (psychological capability and reflective motivation)

There were concerns amongst the participants that there was a lack of understanding of the MECC initiative and it was seen as another ‘thing’ for health and social care workers to do. The participants felt that rebranding MECC and using a name like ‘Chat to Change’ would facilitate organizations to better understand the aim of MECC and how MECC compliments health and social care workers’ roles.

*‘I think chat to change was the, you know, the fact that it shouldn't take a lot of time, it is a chat, it's part of your day, it's worth doing’* (Public Health Manager, P6).

Other ways in making MECC more appealing to organizations included promoting its evidence-base and cost-effectiveness and linking the value of MECC to national and regional priorities such as COVID recovery, personalized care, prevention and health inequalities.

*‘I think you've got to link it to the agenda, that's, you know, that is highly relevant to them, not just say, this is a really good thing to do, you know, and we should all be doing MECC, it needs to be linked to very positive messages around COVID recovery’* (Senior Public Health Lead, P8).

*‘COVID has presented us with an opportunity to promote MECC, and it’s definitely something that we've used in our communications to promote it to sort of say, look, I guess it’s a really good way in, isn’t it because people are thinking about obesity and smoking and the impacts’* (Public Health Doctor, P2).

### Organizational level barriers and facilitators

#### Need for time and resources to plan and implement MECC training (physical opportunity)

MECC trainers felt that they were not granted enough time to plan and implement MECC effectively due to limited time and funding within the organization. This often led to individuals doing multiple jobs to support MECC training, but this felt unattainable in the long term.

*‘I don’t have a team of support, you know, just me and in terms of doing the day to day logistical and the actual doing the doing, basically, I’ve done everything from the strategy through to the tactics, through to the operational elements of all effects, maintaining the website, maintaining MECC link, working with all of these partners, and trying to redesign the training, and all of that, during a global pandemic, has been a really big ask. It’s been very demanding, it’s been very challenging’* (Senior Public Health Worker, P1).

#### Culture change within organizations (reflective motivation)

Participants discussed the overwhelming need for a shift in culture within organizations to increase the perceived value of the MECC initiative alongside other clinical training to support implementation of MECC.

*‘So I think it is about changing that culture around what we value what we commission and what we measure, and how we measure’* (Service lead, P3).

Participants discussed the need to embed MECC into the culture of organizations, but had concerns that they had not witnessed this change within organizations, resulting in a less effective approach.

*‘In order to make it really, really effective until it's really, really, really embedded into the culture of an organisation’* (Role within a commissioning service, P5).

### Individual-level barriers and facilitators

**(1) MECC trainers**

***MECC trainer motivation (reflective motivation)***

The value of the training sessions were deemed an important factor to support MECC trainer motivation. For example, the training gave training agents a Level One Royal Society for Public Health certificate in very brief advice *(this qualification provides learners with an understanding of how social and medical advances have led to improvement in public health)*.

*‘I think people loved the MECC training, when people got on the MECC training. But trying to convince someone who doesn’t know really know what MECC is and doesn’t think it really relates to them, to go on that training was difficult. Whereas actually, if when we just were able to present staff with some short, sharp, practical [tips], this is something you can use this to do. I think that’s when people started to really go with it’* (Service lead, P3).

*‘It’s been great for my sort of professional development, being able to offer that as an extra bit of the training’* (Mental health worker, P7).

**(2) MECC agents**

***Ability to deliver and signpost to appropriate services during MECC conversations (physical opportunity)***

The fear of initiating healthy conversations without the knowledge of where to signpost was raised as a potential barrier. Having access to MECC Link, a website where resources could be accessed by MECC trainers and agents which highlights all local services health workers could signpost to, was seen as a huge asset to the programme, which provided a comprehensive list of updated resources, to support appropriate referrals:

*‘You know, it is not just having that conversation and telling them, it is about where do we go from there, and that’s where MECC link comes in. So it’s been a really, you know, a really sort of robust and cohesive approach to be able to give to people, and it’s so simple’* (Senior Public Health Worker, P1).

## Discussion

### Main findings of the study

This study explored barriers and facilitators to the implementation of MECC, in line with the COM-B model^12^ and provided useful insight which can be translated into practical recommendations for MECC stakeholders, presented in Table 3.

**Table 3:** Recommendation for future MECC initiatives to support implementation

Recommendation	Detail
**1. A national approach to MECC**	It is crucial to first determine the driving force behind MECC to ensure its sustainability. It became apparent that a national approach whereby training materials could be shared between organizations would benefit the local MECC champions. This will provide local leaders the opportunity to embed MECC effectively whilst providing MECC trainers with the tools and capability to deliver the sessions. Furthermore, aiming to ensure that MECC aligns with national and regional priorities such as personalized care, prevention and health inequalities as well as offering advice and support for some of the wider determinants which effect choice such as housing and welfare.
**2. Establishing a co-ordinating role**	To ensure that MECC trainers remain motivated, and that their capabilities are matched to the correct training environment, a new co-ordinating role should be established within each ICS/region/organization. The co-ordinator would be responsible for engaging with MECC trainers and addressing elements of opportunity to deliver, but also the opportunity for MECC agents to engage with the training. In addition, the co-ordinator would be aware of specific areas of regional priorities, interests or prior experiences held by the MECC trainers and would match these up to specific organizations with similar interests or expertise subsequently, facilitating the likelihood of the sessions being delivered. The co-ordinator could also manage MECC resources and MECC link, ensuring the links are up to date so that the platform was reliable and available for anyone using a MECC approach.
**3. Changing the name and promoting the value of MECC**	Addressing the barriers posed by the title MECC and swapping this with other names such as ‘Chat to Change’ is suggested to be a big step forward for those driving MECC. Specifically, this is likely to increase the motivation of organizations looking to adopt MECC but can also provide a different perspective to the healthy conversations held by MECC agents which could yield increased positive outcomes in service users.
**4. MECC training should be considered as part of formal professional development**	MECC training should be considered as formal professional development, with trainers attending and receiving a qualification or certificate to acknowledge their efforts. This would also encourage MECC trainers to take part and help to increase the perceived value of MECC due to its formal qualification, which has previously been found as important when developing new training for professionals.
**5. Expanding and tailoring the resources**	Highlighting services to signpost service users to during conversations will address elements of MECC agent capability but also motivation, as increasing competence could increase intrinsic motivation to have healthy conversations. Providing MECC agents with the ability to identify services and add this into their resources such as using MECClink, a website providing signposting resources in the local area, will aid towards expanding the resources but also tailor it towards the local needs of the organization. However, this would require new roles to be set up, whereby individuals within these roles would be responsible for ensuring that the recommended services meet the required guidelines.

In line with the COM-B model,^12^ we identified social opportunity barriers to implementation. Our findings highlighted a lack of defined roles to implementing MECC and taking ownership of the initiative, which is commonly reported in implementation research.^22^ The importance of having support from national organizations such as Health Education England and NHS England is vital to promote MECC, develop resources and support implementation across the UK to avoid ambiguity. A balance must be achieved between standardized training and the ability to tailor each intervention to the specific context.

Reflective motivation barriers were identified with the value and purpose of MECC being perceived differently amongst individuals. These findings align with previous work by Chisholm et al., which identified that staff from across the breadth of England valued MECC as a public health approach, but that different organizations interpreted it differently, suggesting that the introduction of standardization across MECC training would be beneficial, with a universal approach across the wider health and social care system alongside primary and secondary health care.^6^ In line with the COM-B model, psychological capability needs to be targeted to raise awareness of the MECC programme and its evidence-base.

Further reflective motivation barriers due to beliefs about MECC were raised. One way to address the need for a culture change with regard to moving away from the medical model to a more holistic approach is to include MECC training within undergraduate curriculum for HCPs. A national approach to this has been developed in the Republic of Ireland.^23^ Our findings support previous research which has identified that time constraints, belief about consequences, confidence and social and professional role identity are all factors which influence the application of MECC.^8^^,^^24^ Including MECC training in undergraduate curriculum could facilitate the application of MECC as it will become part of the health practitioners social and professional role identity, but also provide them with the skills and confidence to adopt MECC.^25^

Physical opportunity barriers exist for MECC to be successfully embedded into practice. A clear barrier to health and social care workers initiating MECC was that they did not have anywhere to signpost clients and communities following MECC conversations.^8^^,^^26^ The MECClink offers a practical solution to this barrier, as a website highlights local services health workers can refer services users onto. This is an example of infrastructure and established pathways for referrals to support the implementation of MECC. Wider physical opportunity barriers nationally and within the organization exist around the lack of time and resource to plan and implement MECC. Whilst these barriers have previously been reported,^26^^,^^27^ if MECC was better valued and organizations were aware of the benefits, including cost-effectiveness, then this could better support senior leadership buy-in.

### What is already known about this topic

MECC has the potential to deliver a significant public health resource, at a low cost, across a variety of healthcare contexts to improve public health.^5–7^ Despite this evidence-base and NICE guidelines to implement MECC into practice, the effectiveness of MECC is dependent upon effective implementation of the initiative into health and social care workforces.^10^ Implementation barriers exist, but often research has investigated health and social care workers’ perspectives, with a lack of consideration of the wider perspectives such as those training health and social care workers to deliver MECC.^9^

### What this study adds

This study explores the wider perspectives of implementation of MECC in an ICS. Whilst implementation barriers have been identified, this study highlights several recommendations from the findings to improve implementation of MECC within integrated healthcare settings, and these are set out in Table 3.

### Limitations of this study

This study is not without its limitations; due to the changes in funding and difficulties faced with the implementation of the MECC project as a result of the COVID-19 pandemic, there were missed opportunities prior to the end of the project for further data collection, which could have possibly enhanced the insight and subsequent evaluation of the project. Nevertheless, areas of future research have been highlighted which include evaluating any changes made to the implementation of MECC following the findings of the study. The theoretical underpinnings of the study and analysis of findings will ensure that recommendations arising from this study are more likely to improve the implementation of MECC across organizations.

## Conclusion

MECC has the potential to play an important role in ICSs, to improve public health; however, implementation barriers exist. Several recommendations need to be implemented to encourage the implementation of MECC into ICSs. These include the need for support from national organizations to drive MECC forward, providing MECC trainers with recognized professional development awards and a need to persuade organizations and individuals of the evidence base of MECC.

## Supplementary Material

COREQ_MECC_paper_fdad173

## Data Availability

The data underlying this article will be shared on reasonable request to the corresponding author.

## Notes

**Rebecca Turner,** Research Associate

**Lucie Byrne-Davis,** Professor

**Panayiotis Michael,** Research Assistant

**Nia Coupe,** Lecturer

**Caroline Holtom,** Health Care Public Health Manager

**Cheryl Smith,** Senior Public Health Practitioner

**Jo Hart,** Professor
